# The East Asian-specific *LPL* p.Ala288Thr (c.862G > A) missense variant exerts a mild effect on protein function

**DOI:** 10.1186/s12944-023-01875-3

**Published:** 2023-08-07

**Authors:** Yuepeng Hu, Guofu Zhang, Qi Yang, Na Pu, Kaiwei Li, Baiqiang Li, David N. Cooper, Zhihui Tong, Weiqin Li, Jian-Min Chen

**Affiliations:** 1 Department of Critical Care Medicine, Nanjing Jinling Hospital, Affiliated Hospital of Medical School, Nanjing University, No.305 East Zhongshan Road, Xuanwu District, Nanjing, 210002 Jiangsu China; 2 Institute of Medical Genetics, School of Medicine, Cardiff University, Cardiff, UK; 3 Univ Brest, Inserm, EFS, UMR 1078, GGB, Brest, F-29200 France

**Keywords:** Acute pancreatitis, Gene-environment interaction, Hypertriglyceridemia, Lipoprotein lipase, Missense variant, Alcohol consumption

## Abstract

**Background:**

Lipoprotein lipase (LPL) is the key enzyme responsible for the hydrolysis of triglycerides. Loss-of-function variants in the *LPL* gene are associated with hypertriglyceridemia (HTG) and HTG-related diseases. Unlike nonsense, frameshift and canonical GT-AG splice site variants, a pathogenic role for clinically identified *LPL* missense variants should generally be confirmed by functional analysis. Herein, we describe the clinical and functional analysis of a rare *LPL* missense variant.

**Methods:**

Chinese patients with HTG-associated acute pancreatitis (HTG-AP) were screened for rare nonsense, frameshift, missense or canonical GT-AG splice site variants in *LPL* and four other lipid metabolism-related genes (*APOC2*, *APOA5*, *GPIHBP1* and *LMF1*) by Sanger sequencing. The functional consequences of the *LPL* missense variant of interest were characterized by in vitro expression in HEK-293T and COS-7 cells followed by Western blot and LPL activity assays.

**Results:**

Five unrelated HTG-AP patients were found to be heterozygous for a rare East Asian-specific *LPL* missense variant, c.862G > A (p.Ala288Thr). All five patients were adult males, and all were overweight and had a long history of alcohol consumption. Transfection of *LPL* wild-type and c.862G > A expression vectors into two cell lines followed by Western blot analysis served to exclude the possibility that the p.Ala288Thr missense variant either impaired protein synthesis or increased protein degradation. Contrary to a previous functional study that claimed that p.Ala288Thr had a severe impact on LPL function (reportedly having 36% normal activity), our experiments consistently demonstrated that the variant had a comparatively mild effect on LPL functional activity, which was mediated through its impact upon LPL protein secretion (~ 20% reduced secretion compared to wild-type).

**Conclusions:**

In this study, we identified the East Asian-specific *LPL* c.862G > A (p.Ala288Thr) missense variant in five unrelated HTG-AP patients. We demonstrated that this variant exerted only a relatively mild effect on LPL function in two cell lines. Heterozygosity for this *LPL* variant may have combined with alcohol consumption to trigger HTG-AP in these patients.

## Background

Hypertriglyceridemia (HTG) is a common clinical entity that affects approximately ∼10% of adults worldwide [1, 2]. It is usually diagnosed when the plasma triglyceride (TG) level exceeds 1.7 mmol/L (> 150 mg/dL). As a quantitative trait, HTG has been further divided into three subcategories (i.e., borderline, 1.7–2.3 mmol/L (150–199 mg/dL); high, 2.3–5.6 mmol/L (200–499 mg/dL); and very high, > 5.6 mmol/L (> 500 mg/dL)) by the Expert Panel on Detection, Evaluation, and Treatment of High Blood Cholesterol in Adults [3] and four subcategories (i.e., mild, 1.7–2.3 mmol/L (150–199 mg/dL); moderate, 2.3–11.2 mmol/L (200–999 mg/dL); severe, 11.2–22.4 mmol/L (1000–1999 mg/dL); and very severe, > 22.4 mmol/L (> 2000 mg/dL)) by the Endocrine Society [4]. Mild to moderate HTG increases the risk of cardiovascular disease [5], whereas severe HTG increases the risk of acute pancreatitis (AP) [6]. HTG-induced AP (HTG-AP) accounts for 14.4–41.8% of total AP patients in China but less than 5% in Western countries [7–9]. HTG-AP has a higher morbidity due to complications and poorer outcomes than other types of AP [8].

HTG can be caused by either genetic and/or environmental factors (i.e., excessive alcohol consumption, diet, medications, obesity, etc.) [1, 2, 10, 11]. The 8p22-residing *LPL* gene (OMIM #609,708) encodes lipoprotein lipase, the key enzyme of intravascular metabolism of TG [12]. Subjects with biallelic *LPL* variants that result in the complete or almost complete loss of LPL function develop type I hyperlipoproteinemia or familial chylomicronemia syndrome, a rare autosomal recessive metabolic disorder that usually occurs in childhood and is characterized by severe HTG with episodes of abdominal pain, recurrent AP, eruptive cutaneous xanthomata and hepatosplenomegaly [13]. To date, hundreds of *LPL* variants have been identified through the study of subjects with familial chylomicronemia syndrome, simple HTG or HTG-associated/induced diseases (see the Human Gene Mutation Database (HGMD; http://www.hgmd.org) [14]). While the pathogenicity of nonsense, frameshift and canonical GT-AG splice site variants is often self-evident, that of missense variants often has to be experimentally determined. Herein, we describe our findings from the clinical and functional analysis of a rare East Asian-specific *LPL* missense variant, c.862G > A (p.Ala288Thr).

## Methods

### Patients

Five unrelated Chinese patients with HTG-AP participated in this study. The diagnosis of AP was made according to the revised Atlanta 2012 Classification [15] and required at least two of the following clinical features: (i) typical abdominal pain associated with the pancreas, (ii) an elevated level of serum lipase or amylase at least three times the normal upper limit, and (iii) characteristic findings of AP upon imaging examinations. The diagnosis of HTG-AP was made when the patient had a serum TG level of ≥ 11.3 mmol/L (1000 mg/dL) or between 5.65 and 11.3 mmol/L (500–1000 mg/dL) with the presence of emulsion plasma at AP onset [16, 17]. The diagnosis of fatty liver disease was made according to typical imaging features (i.e., a low-density hepatic parenchyma on computed tomographic scanning) [18, 19]. History of alcohol consumption was described in terms of duration (years) coupled with the estimated alcohol intake quantity assessed in grams (g) per week according to the type, volume and frequency of the alcoholic products imbibed.

### Variant identification and nomenclature

Genomic DNA was extracted from blood using the Gentra Puregene Blood Kit (Qiagen, Dusseldorf, Germany) according to the manufacturer’s instructions. All coding regions and flanking intronic regions of the *LPL* gene and four other lipid metabolism-related genes (i.e., *LMF1* (lipase maturation factor 1, OMIM #611,761), *GPIHBP1* (glycosylphosphatidylinositol-anchored high density lipoprotein-binding protein 1, OMIM #612,757), *APOA5* (apolipoprotein A-V, OMIM #606,368), and *APOC2* (apolipoprotein C-II, OMIM #608,083)) were analyzed by Sanger sequencing as previously described [20–23]. Only rare nonsense, frameshift, missense and canonical GT-AG splice site variants were considered. Rare variants were defined as having an allele frequency of < 0.01 [24] by reference to the global population data in the Genome Aggregation Database (gnomAD; https://gnomad.broadinstitute.org/). Variant nomenclature followed Human Genome Variation Society (HGVS) recommendations [25], with NM_000237.3 being used as the reference *LPL* mRNA sequence.

### *In silico* analyses

The evolutionary conservation of the *LPL* p.Ala288 residue was evaluated in the context of a multiple species protein sequence alignment and by means of Genomic Evolutionary Rate Profiling (GERP; http://mendel.stanford.edu/sidowlab/downloads/gerp/index.html), phastCons46way (https://genome.ucsc.edu/cgi-bin/hgTrackUi?db=hg19&g=cons46way), phastCons100way (https://genome.ucsc.edu/cgi-bin/hgTrackUi?db=hg19&g=cons100way), phyloP46way [26, 27], and phyloP100way [26, 27].

The three-dimensional (3D) structures of wild-type and p.Ala288Thr LPL proteins were predicted using PyMOL software [28].

Pathogenicity predictions of the *LPL* p.Ala288Thr missense variant by Polyphen and SIFT were taken from the gnomAD website (https://gnomad.broadinstitute.org/; as of 11 May 2023).

### Plasmid construction, cell culture and transfection

Human wild-type and c.862G > A *LPL* cDNAs were synthesized and cloned into the pcDNA3.1 vector by GenScript (Nanjing, China). Plasmid construction was confirmed by Sanger sequencing. The HEK-293T (ATCC, CRL-3216) and COS-7 (ATCC, CRL-1651) cell lines, neither of which exhibited endogenous LPL expression, were cultured in Dulbecco’s modified Eagle’s medium (DMEM) with 10% fetal bovine serum (FBS) and 1% penicillin‒streptomycin (PS) at 37 °C in a humidified chamber supplemented with 5% CO_2_. Six-hour transient transfections were performed in 6-well plates (Corning; product number 354,573) using Lipofectamine 3000 (Thermo Fisher Scientific Inc.; product number L3000015) according to the manufacturers’ instructions. For experiments with respect to protein synthesis, the transfected cells were changed to DMEM with 2% FBS and cultured for an additional 48 h before the cells were collected for analysis. Where LPL secretion and enzyme activity were to be measured, heparin treatment was performed by replacing the medium with 500 µL heparin-DMEM mixture (ratio of heparin and DMEM was 8:500) and culturing for another 30 min; both the cells and media were then collected for analysis.

### Analysis of LPL mass and activity

The cell medium was centrifuged at 4 °C for 10 min at 12,000 rpm to remove cells and debris, and the supernatant was collected and stored at -80 °C for further analysis. The transfected cells were harvested and treated with RIPA lysis buffer (Beyotime, China) for 30 min. The cell suspension was centrifuged at 4 °C for 10 min at 12,000 rpm, while the lysates and supernatants were collected and stored at -80 °C. Western blotting was performed to analyze the expression of LPL protein in both the cell medium and lysate. The antibodies (all from Santa Cruz Biotechnology (Shanghai)) and antibody dilutions used in this study were as follows: primary rabbit LPL antibody (product number 73,646), 1:200; primary mouse GAPDH antibody (product number 47,724), 1:5000; secondary anti-rabbit IgG-HRP (product number 2357), 1:2000; and secondary anti-mouse IgG-HRP (product number 2004), 1:5000. Analysis of band intensity was performed by means of ImageJ software. Analysis of LPL activity in the cell medium was performed as previously described [29]. All experiments were repeated at least 3 times independently. The results of Western blotting and LPL activity analysis are shown as the mean ± standard deviation (SD) and were analyzed by the SPSS 25.0 software package (IBM Analytics, Armonk, NY). A probability (*P* value) of less than 0.05 was defined as being statistically significant.

## Results

### Clinical features of the five patients

Demographic baselines and clinical features of the five patients are summarized in Table 1. Notably, all five patients were adult males, overweight (body mass index of > 25 but < 30), were currently suffering from HTG-AP after a high-fat diet and/or alcohol consumption, developed severe or critical AP in accordance with the determinant-based classification, had severe or very severe HTG at AP disease onset, and had a history of HTG and a long history of alcohol consumption. Moreover, four of the five patients had a fatty liver, and three patients had experienced previous episodes of AP.

**Table 1:** Demographic baselines and clinical features of the five HTG-AP patients studied

Variable	Patient #1	Patient #2	Patient #3	Patient #4	Patient #5
**Age (years; at the current disease onset)**	41	29	57	42	59
**Sex**	Male	Male	Male	Male	Male
**Body mass index (kg/m** ^**2**^ **)**	28.07	27.17	25.06	28.72	26.81
**Putative triggering factor(s)**	HFD & Alcohol (~ 30 g)^a^	HFD	HFD & Alcohol (~ 40 g) ^a^	Alcohol (~ 80 g) ^a^	Alcohol (~ 80 g) ^a^
**TG at AP onset (mmol/L)**	30.1	22.7	13.1	18.8	96.7
**APACHE II score**	9	7	17	30	21
**Severity of AP**					
RAC	Severe	Moderate severe	Severe	Severe	Severe
DBC	Severe	Severe	Critical	Critical	Critical
**Complications**					
Acute respiratory distress syndrome	No	No	Yes	Yes	Yes
Acute kidney injury	Yes	No	Yes	Yes	Yes
Abdominal compartment syndrome	No	No	No	Yes	No
Infected pancreatic necrosis	No	Yes	Yes	Yes	Yes
Abdominal hemorrhage	No	No	No	No	Yes
**Length of hospital stay (days)**	7	18	115	48	42
**Length of ICU stay (days)**	6	5	58	48	42
**Outcomes**	Survival	Survival	Survival	Death	Death
**Medical histories**					
AP	No	Yes	Yes	Yes	No
Number of previous AP episodes	0	1	2	1	0
Age of the first AP episode (years)	41	27	52	40	59
Diabetes mellitus (prior treatment)	Yes, 3 years (Metformin, 1000 mg/day)	No	No	No	No
HTG	Yes, 15 years	Yes, 3 years	Yes, 5 years	Yes, 10 years	Yes, 20 years
Hypertension	Yes, 5 years	No	Yes, 8 years	No	Yes, 10 years
Fatty liver	Yes, 10 years	Yes^b^	Yes^b^	No	Yes^b^
Smoking	No	No	Yes, 30 years (30 cigarettes/day)	Yes, 15 years (40 cigarettes/day)	Yes, 30 years (30 cigarettes/day)
Alcohol consumption (duration, average weekly intake quantity of ethanol)	Yes, 15 years (~ 200 g/week)	Yes, 5 years (~ 80 g/week)	Yes, 30 years (~ 240 g/week)	Yes, 20 years (~ 400 g/week)	Yes, 30 years (~ 160 g/week)

^a^Amount of alcohol consumed immediately prior to the onset of the current HTG-AP

^b^The duration of the previous disease was unclear

Abbreviations: AP, acute pancreatitis; APACHE II score, Acute Physiologic Assessment and Chronic Health Evaluation Scoring System II [40]; HFD, high-fat diet; HTG, hypertriglyceridemia; ICU, intensive care unit; TG, triglyceride; RAC, revised Atlanta classification [15]; DBC, determinant-based classification [41]; ICU, intensive care unit

### All five patients were heterozygous carriers of the LPL c.862G > A (p.Ala288Thr) missense variant

All five patients were found to carry a rare heterozygous *LPL* missense variant, c.862G > A (p.Ala288Thr). Here, it should be emphasized that (i) these five patients represent the totality of patients carrying a heterozygous *LPL* c.862G > A variant among our 492 HTG-AP patients analyzed from January 2020 to December 2022 and (ii) *LPL* c.862G > A was the only variant we found in these five patients, in terms of rare nonsense, frameshift, missense or canonical GT-AG splice site variants in the five primary HTG-related genes analyzed (i.e., *LPL*, *LMF1*, *GPIHBP1*, *APOA5* and *APOC2*).

In gnomAD, *LPL* c.862G > A is listed as having an allele frequency of 0.0007611 (14/18,394) in the East Asian population but is absent from all other assigned populations, including South Asians. A literature search revealed that the c.862G > A variant has been previously reported in five studies; all carriers were of either Chinese [30–33] or Japanese origin [34]. Therefore, *LPL* c.862G > A is a rare East Asian-specific missense variant.

The c.862G > A allele was significantly associated with HTG-AP in Chinese patients using gnomAD East Asians as controls (5/984 (0.508%) vs. 14/18,394 (0.076%); odds ratio (OR) = 6.676, 95% confidence interval: 2.410-18.498; *P* = 0.002).

### Functional characterization of the *LPL* p.Ala288Thr missense variant in two cell lines

**Fig. 1:**
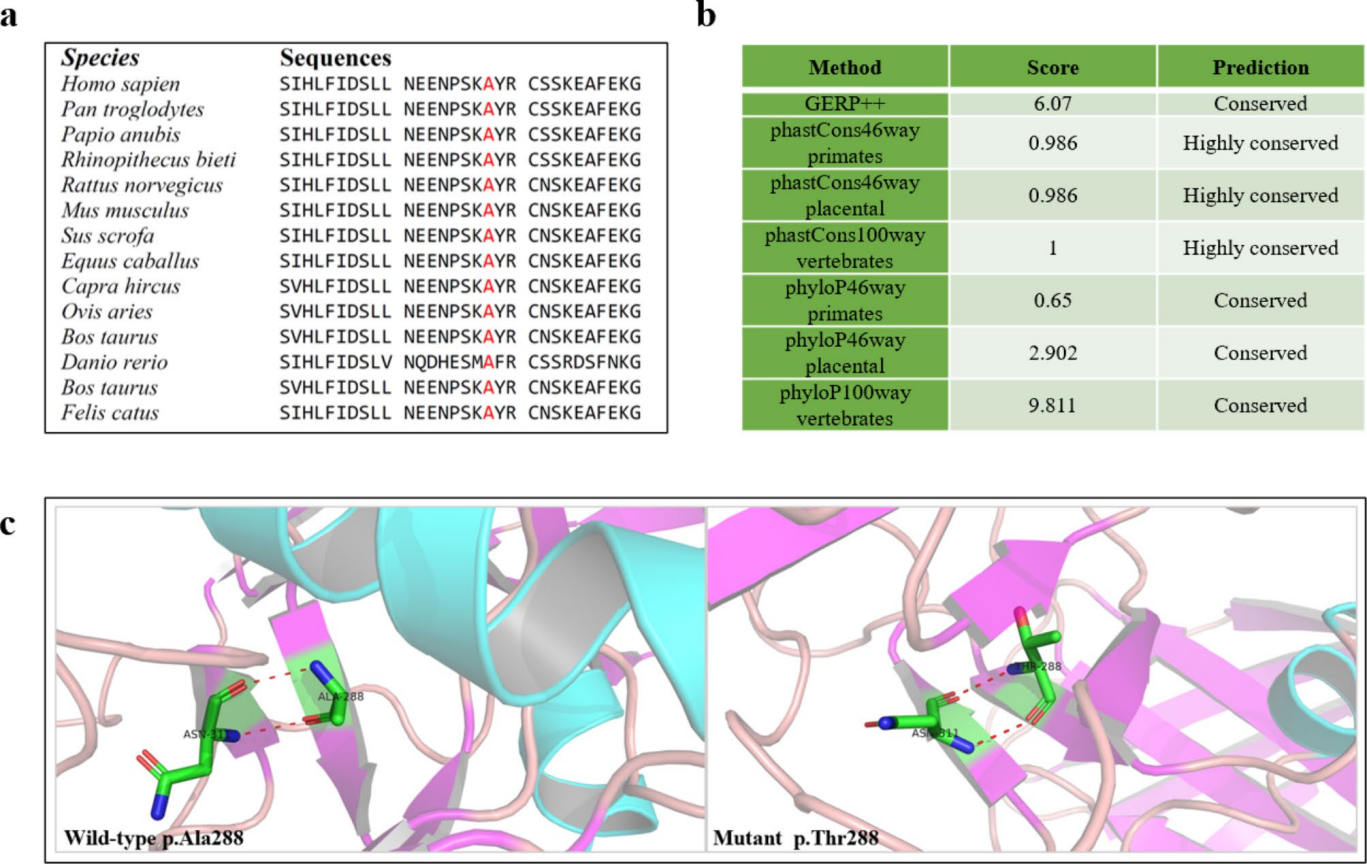
*In silico* analyses pertaining to the *LPL* p.Ala288Thr variant. (**A**) Alignment of partial vertebrate LPL amino acid sequences spanning the p.288 site. (**B**) Conservation scores of the LPL p.288 site as predicted by the indicated programs. (**C**) Predicted partial 3D structures of the wild-type (p.Ala288) and mutant (p.Thr288) LPL proteins

The LPL p.Ala288 residue is evolutionarily conserved (Fig. 1A, B), and the p.Ala288Thr missense variant appears to modify LPL protein structure (Fig. 1C), suggesting that p.Ala288Thr could significantly affect LPL function. Moreover, p.Ala288Thr has been predicted to be “probably damaging” by Polyphen and “deleterious” by SIFT. Furthermore, and most importantly, the mass and activity of the LPL p.Ala288Thr mutant protein secreted into the media of transfected COS-1 cells have been previously reported to be 67% and 36% those of the wild-type [30]. However, whether the reduced mass and activity were related to reduced protein synthesis and/or to increased protein degradation was unclear. This prompted us to perform a new functional characterization of the LPL p.Ala288Thr missense variant.

**Fig. 2:**
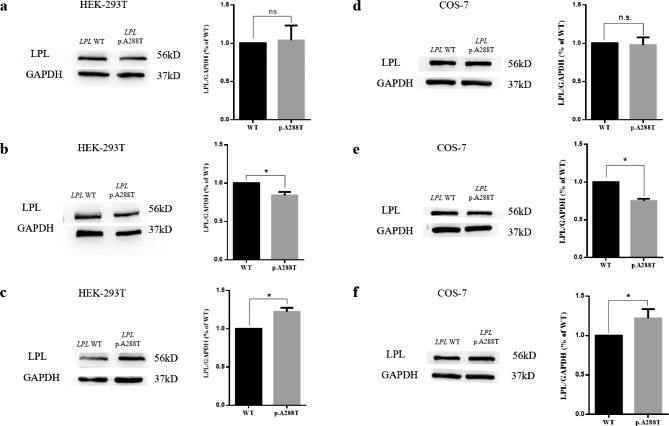
Functional analysis of the *LPL* p.Ala288Thr missense variant. (**A**) Western blot (WB) analysis of LPL expression in transfected HEK-293T cells without heparin. (**B**) WB analysis of postheparin LPL expression in cell medium from HEK-293T cells. (**C**) WB analysis of postheparin LPL expression in cell lysates from HEK-293T cells. (**D**) WB analysis of LPL expression in transfected COS-7 cells without heparin. (**E**) WB analysis of postheparin LPL expression in cell medium from COS-7 cells. (**F**) WB analysis of postheparin LPL expression in cell lysates from COS-7 cells. LPL, lipoprotein lipase; WT, wild-type; p.A288T, *LPL* p.Ala288Thr; ns, not significant; * *P* < 0.05

**Fig. 3:**
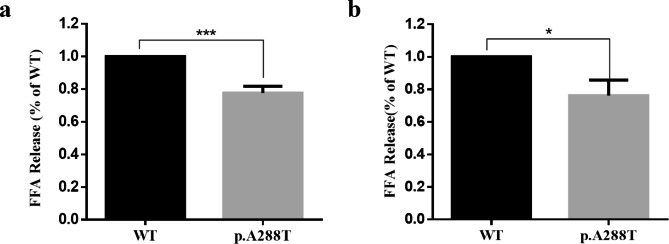
Relative postheparin LPL activity in the medium of transfected cells. (**A**) Free fatty acid (FFA) level of cell medium from HEK-293T cells. (**B**) FFA level of cell medium from COS-7 cells. The results are shown as the mean ± SD from three independent transfections, and all assays were performed in three replicates. LPL, lipoprotein lipase; WT, wild-type; p.A288T, *LPL* p.Ala288Thr; *** *P* < 0.001

We initially performed cell transfection experiments in HEK-293T cells and employed Western blotting to measure the relative levels of the expressed wild-type and mutant LPL proteins in the transfected cells (without heparin treatment), but no significant differences were evident (Fig. 2A). This essentially excluded the possibility that the mutant LPL protein was subject to reduced synthesis or increased degradation. We then attempted to replicate the findings of the Ma study [30]. Unexpectedly, we found that the p.Ala288Thr missense variant exerted only a relatively mild effect on LPL secretion (Fig. 2B, C), which was concordant with its similarly mild impact on LPL activity (Fig. 3A). To confirm or refute these findings, we repeated the experiments in COS-7 cells (Figs. 2D, E and F and 3B) and obtained comparable results to those produced in HEK-293T cells.

In short, our experiments in two different human cell lines demonstrated that the p.Ala288Thr missense variant resulted in a consistent ~ 20% reduction in LPL protein secretion with a concomitant ~ 20% reduction in LPL activity.

## Discussion

Subsequent to identifying the rare and East Asian-specific *LPL* c.862G > A (p.Ala288Thr) missense variant in five unrelated Chinese patients with HTG-AP, we re-evaluated the functional effect of this missense variant. In two cell lines (HEK-293T and COS-7), we found that the p.Ala288Thr variant had no effect on protein synthesis and/or degradation, a finding not previously reported. Moreover, contrary to the results of a previous study [30], we found that the p.Ala288Thr missense variant had only a mild effect on LPL functional activity, mediated through its impact upon LPL protein secretion (~ 20% reduced secretion compared to wild-type). The precise reasons for the discrepancy with previously published results are unclear but may be related to differences in experimental conditions/technical procedures resulting from the intervening timespan of nearly 30 years between the two studies.

The p.Ala288Thr missense variant was first identified in a Chinese female with HTG-AP during pregnancy [30]. This patient also carried a second *LPL* missense variant, p.Leu279Arg. The two missense variants were shown to be located in *trans* by means of colony sequencing PCR-amplified exon 6 products from the *LPL* gene. Since p.Leu279Arg was experimentally demonstrated to be a null variant [30], any residual in vivo LPL activity detectable in the compound heterozygous patient should have been attributable to LPL synthesis from the partially functional p.Ala288Thr allele. In this regard, this patient was reported to have 25% plasma LPL activity compared to controls [30]. This level of in vivo activity was slightly higher than the 18% (36%/2) activity of the p.Ala288Thr mutant as determined in vitro in the Ma study [30] but lower than the 40% (80%/2) activity of the p.Ala288Thr mutant determined in our current study. However, the measurement of in vivo LPL activity (with the exception of complete or almost complete loss of activity) may be confounded by many factors, including genetic variants, diet, lifestyle and environment. Therefore, considerable caution should be exercised when attempting to correlate in vivo LPL activity with in vitro determined LPL activity for any variant of interest.

Our functional analysis data suggest that the catalytic activity of the LPL protein would not be impaired by the substitution of alanine by threonine at amino acid position 288. Some other *LPL* missense variants (e.g., p.Ser325Arg [35] and p.Cys445Tyr [36] have also been reported to affect LPL secretion but not the catalytic activity of the LPL protein. Further examples of functionally characterized *LPL* missense variants would improve our understanding of LPL protein structure and function.

Gene‒environment interactions play a vital role in the pathogenicity of many diseases [37], including HTG-AP [20, 21]. In a given disease/gene context, a variant associated with a mild functional effect may have to interact synergistically with other genetic and environmental factors for it to come to clinical attention, whereas this is much less likely to be the case for variants with a more severe functional impact. Our functional analyses in two cell lines clearly demonstrated that *LPL* p.Ala288Thr is a mild variant in terms of its functional effect. Interestingly, all five heterozygous p.Ala288Thr patients were overweight (body mass index > 25.0), and all had a long history of alcohol consumption. Moreover, four of the 5 patients had been drinking alcohol to excess immediately prior to the onset of HTG-AP (Table 1). Alcohol consumption is closely associated with plasma TG levels at the population level; it may well be that the combination of alcohol consumption and underlying genetic risk factors for HTG hastens or even triggers the development of severe HTG [2, 38].

A recent meta-analysis of 127 studies performed between 2012 and 2022 indicated that HTG-AP had not only the highest risk for a nonmild (moderately severe and severe) condition but also a much higher mortality rate than alcoholic AP (OR = 1.72) and biliary AP (OR = 1.50) [39]. To minimize the risk of HTG-AP, it is important to maintain a healthy lifestyle and to avoid environmental risk factors, especially if one is a carrier of pathogenic variants in the *LPL* gene or other HTG-related genes.

The strengths of our study were that (i) the cell transfection experiments were performed in two cell lines (HEK-293T and COS-7) and under two conditions (with and without heparin treatment) and (ii) the functional effects of the p.Ala288Thr missense variant were analyzed by both Western blot and activity analyses. The limitations of our study were that (i) we were unable to obtain in vivo LPL mass and activity values from any patients and (ii) we did not include common genetic variants for analysis.

## Conclusions

In this study, we identified the infrequent East Asian-specific *LPL* c.862G > A (p.Ala288Thr) missense variant in 5 unrelated HTG-AP patients. Using gnomAD East Asians as controls, c.862G > A confers a significant risk for HTG-AP in Chinese patients (OR = 6.676). In two different cell lines, we demonstrated that the p.Ala288Thr missense variant had only a mild effect on LPL secretion. We highlighted an association of this *LPL* variant with alcohol consumption in all five HTG-AP patients.

## Data Availability

All supporting data are available within the article.

## Abbreviations

AP: acute pancreatitis
APACHE II: Acute Physiologic Assessment and Chronic Health Evaluation Scoring System II
APOA5: apolipoprotein A-V
APOC2: apolipoprotein C-II
DBC: the determinant-based classification
GPIHBP1: glycosylphosphatidylinositol-anchored high density lipoprotein-binding protein 1
HFD: high-fat diet
HGMD: Human Gene Mutation Database
HGVS: Human Genome Variation Society
HTG: hypertriglyceridemia
HTG-AP: hypertriglyceridemia-related acute pancreatitis
ICU: intensive care unit
LMF1: lipase maturation factor 1
LPL: lipoprotein lipase
OR: odds ratio
RAC: the revised Atlanta classification
SD: standard deviation
TG: triglyceride
